# Effect of segmentation of k-space in SSFP flow artifacts

**DOI:** 10.1186/1532-429X-17-S1-P53

**Published:** 2015-02-03

**Authors:** Saeid Ahmadinia, Abbas N Moghaddam

**Affiliations:** 1 BME, Tehran Polytechnic, Tehran, Iran, the Islamic Republic of; 2 UCLA, Los Angeles, CA, USA

## Background

Steady State Free Precession is a fast technique with high contrast between myocardium and blood in cardiovascular MR imaging. According to the time consuming data acquisition and short cardiac cycle (almost 0.9 second), data is obtained in several heartbeats depending on the number of frames and K-space size. The K-Space is divided to segments and each segment is acquired in one cardiac cycle. However, spins in the flowing blood do not experience enough pulses to reach the steady state condition. As a result, shadows called flow artifacts appear in images. In this study, we investigate the flow artifacts and in particular, how the number of frames (or segments) and in-plane velocity affect them.

## Methods

We utilized a Bloch simulator to consider a 3D space of moving spins; the magnetization and position of spins are updated after each pulse excitation.

The simulated motion is a sinusoidal in-plane flow, moving from left to right with a parabolic profile, resembling a cardiac cycle of 1 second. In addition, a through-plane flow brings unsteady fresh spins to the imaging slice. The through-plane velocity is high enough to bring fresh spins to the slice after each excitation.

## Results

**Figure 1:**
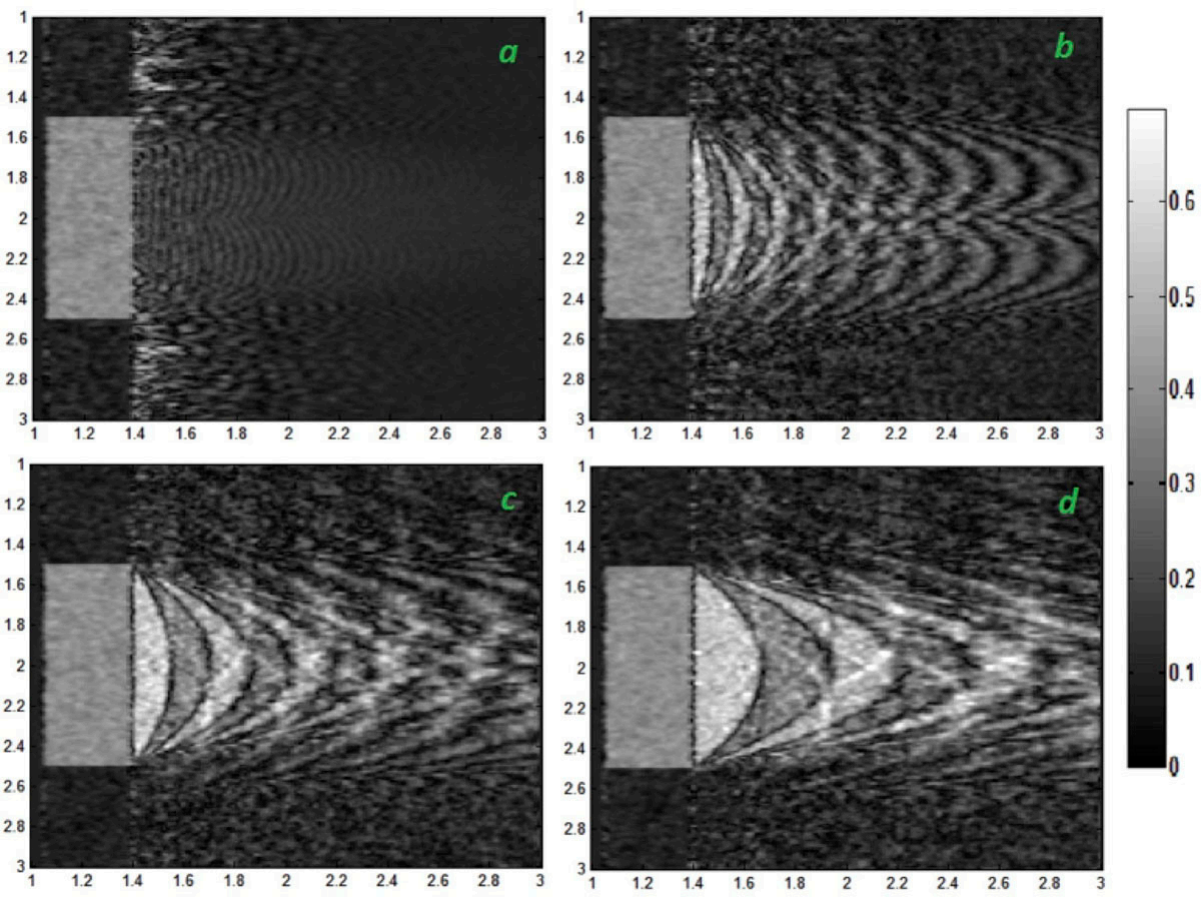
The 15^th^ frame among 20 frames for in-plane velocities of: a. 5 cm/s b. 20 cm/s c. 40 cm/s d. 70 cm/s; The artifact severity increases with the in-plane velocity.

The in-plane velocity makes spins to move while data acquisition is being performed. Therefore, it could play an important role in artifact severity. As Fig. 1 illustrates, the in-plane velocity is proportional to the artifact since spins move more quickly.

**Figure 2:**
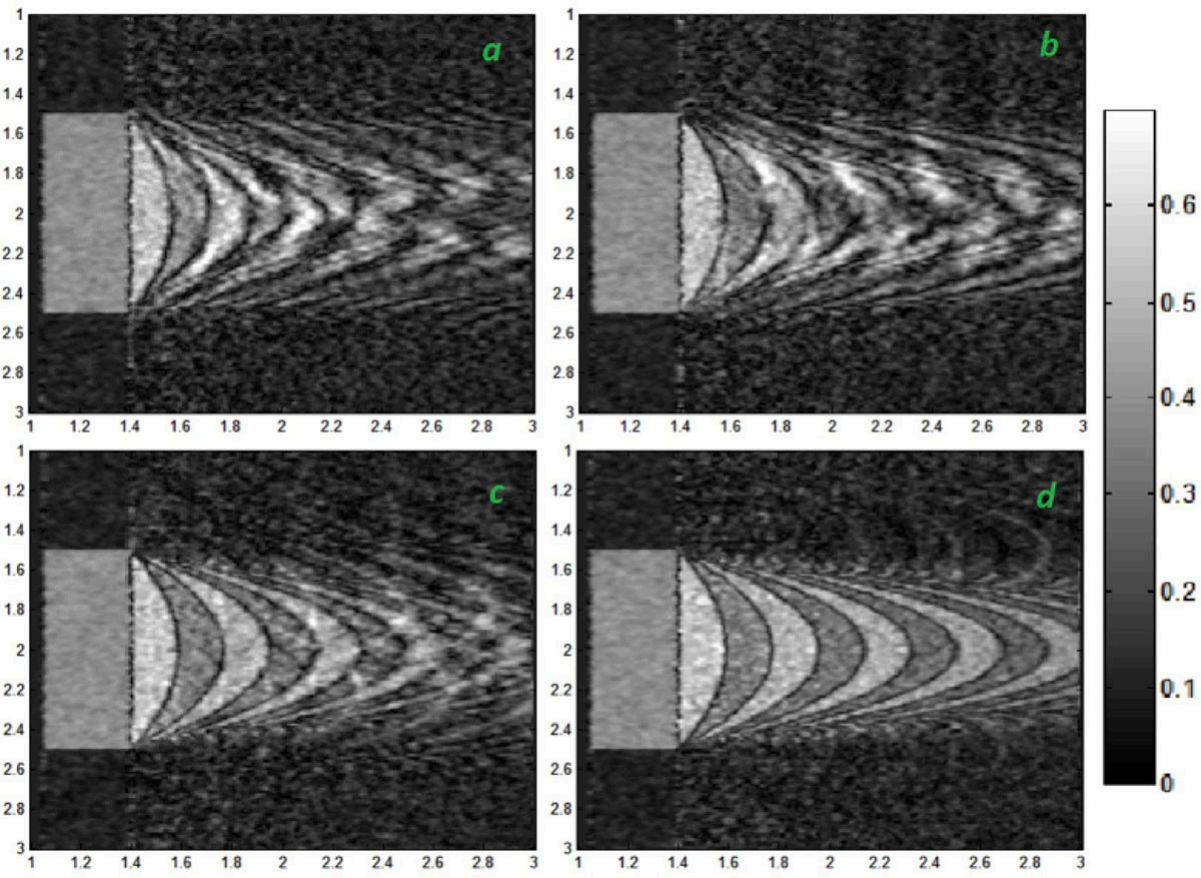
The in-plane peak velocity is 40 cm/s, the simulation was done while the number of frames is a. 5 frames, b. 10 frames, c. 20 frames, d. 50 frames.

The number of k-space rows filled in each period is inversely proportional to the number of required frames. The larger the segments of k-space, the stronger the artifacts will be. In fact, large segments lead k-space to record more variations, so more artifacts will appear. (Fig. 2)

## Conclusions

Fluid motions prevent spins from reaching steady state. Therefore, some artifacts appear. The severity of the artifacts depends on factors like the in-plane velocity and the number of frames acquired.

The number of frames determines the number of rows filled in each period. In fact, the number of frames is inversely proportional to the number of rows filled in each excitation. From the other side, less row numbers acquired in each cardiac cycle leads to less flow artifact since fewer variations happen during the K-space formation. On the other hand, fewer in-plane velocities also have the same effects on contributing the artifact.

In Summary, the in-plane velocity and the number of frames both could affect the artifact severity. Both of them contribute to add an extra phase while segments are being formed. The added phase in K-space domain leads to develop some spatial shift known as artifacts in images.

## Funding

N/A.

